# Maintenance dynamics in digital health public goods: a quasi-experimental study of issue resolution during the 2025 USAID funding disruption

**DOI:** 10.1093/oodh/oqag035

**Published:** 2026-09-05

**Authors:** Jinyou Sheng, Amy Finnegan

**Affiliations:** Duke Global Health Institute, Duke University, Trent Hall, 310 Trent Drive, Durham, NC, 27710, United States; Duke Global Health Institute, Duke University, Trent Hall, 310 Trent Drive, Durham, NC, 27710, United States

**Keywords:** digital health public goods, open-source software sustainability, funding shocks, bug resolution efficiency, organizational resilience

## Abstract

Open-source digital public goods play a growing role in point-of-care health service delivery in low- and middle-income countries. Many of these systems are developed and maintained by organizations that rely heavily on donor funding, making their long-term sustainability vulnerable to funding instability. The January 2025 U.S. Agency for International Development funding freeze created a rare natural experiment to observe how different organizations maintaining these systems respond under external funding shocks. Using 7922 issues from eight repositories created between June 2023 and June 2025, a DistilBERT model was fine-tuned to classify bug severity. Organizations were categorized into three funding-based models: donor-driven (primarily grant-funded), revenue-driven (sustained through commercial or internal investment), and hybrid (combining grants and revenue). Interrupted time series and Cox proportional hazards models were then applied to evaluate changes in bug-resolution efficiency. Revenue-driven organizations did not exhibit a statistically significant immediate level change but experienced significant deterioration in the post-intervention period. Donor-driven organizations exhibited no substantial changes following the intervention, maintaining stable baseline levels. Hybrid organizations showed resilience to the immediate funding shock, with a gradual decline in bug resolution as time went on after the shock. These findings suggest that the funding shocks function as a stress test for digital public goods and highlight the resilience advantages of various governance models.

## Introduction

Free and Open-Source Software (FOSS) has become central to digital transformation efforts in global public sector service delivery. As a software model that makes source code openly available and allows users to legally use, modify, and redistribute it, FOSS is widely recognized as a foundational form of digital public infrastructure. Studies suggest that open-source components are present in ~96% of codebases and account for up to 90% of commercial software stacks (Osborne et al. 2024). Public and private donors have increasingly promoted open-source platforms as a way to reduce costs, enhance interoperability, and avoid vendor lock-in (DIAL 2020).

Despite these advantages, the long-term sustainability of FOSS remains a persistent concern. Many open-source projects depend on volunteer labor, fragmented governance structures, and unstable funding streams (FOSS United 2024; El-Komboz and Goldbeck 2025). These structural fragilities raise concerns about the capacity of such projects to sustain ongoing maintenance, particularly as FOSS increasingly supports mission-critical public systems. This issue is especially salient given that FOSS components now underpin essential infrastructures ranging from cybersecurity systems to national health information platforms, where maintenance quality directly affects system reliability and long-term resilience (Osborne et al. 2024; Champion and Hill 2021).

In the context of global digital health, these sustainability challenges are further shaped by the sector’s distinctive funding structure. In practice, organizations maintaining these systems operate under different funding models, including donor-driven models reliant on grants, revenue-driven models sustained through commercial or internal investment, and hybrid models that combine both. Unlike purely volunteer-driven open-source ecosystems, many FOSS initiatives in this space are embedded within global health financing architectures, where external donors play a central role in supporting development, deployment, and maintenance activities. Among these donors, the United States, primarily through the U.S. Agency for International Development (USAID), has historically been one of the largest and most influential funders of health initiatives in low- and middle-income countries (LMICs), contributing approximately $12–13 billion annually to global health with an increasing focus on open source digital public infrastructure to support program results (KFF 2025). As a result, many FOSS projects, as well as the organizations that maintain them, are directly or indirectly dependent on U.S. foreign assistance.

Such dependencies become particularly visible during periods of funding disruption. In January 2025, the U.S. Office of Management and Budget (OMB) announced a temporary suspension of all new federal grants and cooperative agreements, creating immediate uncertainty across the public-interest technology landscape (The White House 2025). This pause was subsequently extended, resulting in a prolonged disruption to funding flows. Given the central role of U.S. funding in this ecosystem, this policy shift constituted a system-wide shock that affected not only direct recipients but also downstream implementing partners and organizations responsible for maintaining open-source digital public goods.

This study examines how the USAID funding freeze affected the maintenance of FOSS global digital health software. Bug resolution efficiency is used as an indicator of operational stability. Resolution patterns are compared across organizational types defined by their underlying funding models and across severity levels standardized using transformer-based text classification. By estimating changes in both the level and trend of bug resolution before and after the funding freeze, the analysis evaluates how different organizational structures shape the resilience of FOSS projects.

## Data and methods

### Study design

This study adopts a quasi-experimental interrupted time series (ITS) design combined with survival analysis to examine bug resolution efficiency in open-source digital public goods for point-of-care service delivery in health facilities and communities. The analytic window was restricted to June 2023–June 2025 to ensure comparability across projects initiated at different times and to exclude the influence of the COVID-19 period which likely had an impact on operational efficiency.

### Data sources and sample selection

This study focused on open-source digital public goods related to point-of-care service delivery by community health workers and facility-based care (electronic health records, EHRs) identified from the Digital Public Goods Alliance (DPGA) registry. The DPGA is a UN-endorsed, multi-stakeholder initiative that facilitates the discovery, development, and deployment of open-source digital technologies to support the achievement of the Sustainable Development Goals (SDGs). From an initial pool of digital public goods in the DPGA registry (n = 51), 12 initial candidate applications were identified, then four were excluded due to having fewer than 150 publicly available GitHub issues. The final analytic sample comprised eight applications representing three distinct revenue architectures and governance structures: revenue-driven, donor-driven, and hybrid models combining revenue and grants. Revenue-driven organizations often rely on internal corporate investment; for example, Open Health Stack was developed as an open-source initiative led by Google in collaboration with USAID as the first model of a public-private partnership in global digital health. Donor-driven organizations are frequently supported by public and philanthropic grants; for example, the OpenMRS platform has received funding or support from donors such as the Rockefeller Foundation, the U.S. Centers for Disease Control and Prevention, and the World Health Organization (WHO). Hybrid organizations typically combine donor grants with commercial revenue from implementation services and software deployment (see Table 1).

**Table 1:** Classification of organizational types of digital public goods analyzed.

Category	Company type	Description
Revenue-driven	Private Company	Privately owned companies that develop and maintain commercial health technologies. Example app: Open Health Stack (by Google).
Donor-driven	International Non-governmental Organization	Donor-driven organizations operating across countries and focusing on service delivery, advocacy, or humanitarian support. Example apps: OpenMRS-core (by the OpenMRS community), openmrs-module-bahmniapps (by Bahmni), Community Health Tookit (by Medic), and iHRIS (by IntraHealth International).
Hybrid (revenue and donor)	Hybrid organizations	Mission-driven organizations that use commercial strategies to achieve social or environmental goals. Example apps: CommCare (by Dimagi), E-Heza (by TIP Global Health), and opensrp-client-core (by OpenSRP).

The revenue-driven category is represented by a single case (Open Health Stack by Google) to serve as an illustrative example of a revenue-driven model, where engagement in open-source digital health is sustained through internal strategic investment.

All digital public goods included in this study have historically received support from USAID or from implementing partners funded through USAID digital health programs. Although repository-level funding information for January 2025 could not be consistently verified for all projects, these platforms operate within a global health implementation ecosystem that is heavily dependent on foreign aid. Disruptions in foreign assistance likely affected not only direct funding flows but also downstream activities among implementing partners, ministries of health, and deployment teams that rely on these systems. Accordingly, this study treats all included projects as affected by the funding freeze, even without confirmed repository-level funding in that specific month.

### Variable construction and preprocessing

GitHub issue and pull request metadata were compiled from the eight selected repositories. Key variables included the issue or pull request title, opening and closing date for each issue, contributor-assigned tags, and repository name. Text data (titles and bodies) were concatenated, converted to lowercase, stripped of non-alphanumeric characters, and stemmed using the Porter Stemmer (a process that reduces words to their root forms, e.g. ‘running’ becomes ‘run’) to reduce lexical variation. To capture weekly maintenance dynamics, three key metrics were constructed: the weekly number of new issues reported, the count of issues resolved each week, and the active issue load, measured as the total number of issue-days (i.e. the sum of overlapping days that issues remain open within each week). The closure rate is defined as the percentage of active issues resolved within a given week, enabling size-adjusted comparisons across organizations. In standard workflows, issues are typically closed only after fixes are implemented and tested or merged. Although some closures are administrative (e.g. duplicates or invalid reports), inspection of issue threads and pull requests indicates that most reflect actual fixes. Therefore, issue closure is used as a proxy for bug resolution in this study.

### Severity classification

To classify bug severity, a DistilBERT-based model was trained by adapting the data preprocessing pipeline, feature construction approach, and overall workflow from the open-source *bugClassificationSystem* repository. Training was performed on two publicly available GitHub bug-classification datasets with manually annotated severity labels. Text inputs were tokenized with *DistilBertTokenizerFast* (maximum length = 128 tokens, with padding and truncation), and the data were split into training (80%) and testing (20%). Model training ran for three epochs with a batch size of eight, using the Trainer API with accuracy as the evaluation metric.

Performance was evaluated on the held-out test set using precision, recall, and weighted F1-score, a harmonic mean of precision and recall that provides a balanced measure of a model’s accuracy, particularly useful when dealing with imbalanced data (Yacouby and Axman 2020). Two model versions were compared: one retaining stop words and another removing them. Stop words, the common words with limited semantic meaning (e.g. the, and, is), were removed to reduce noise. The stop-word–removed model outperformed the retained version, achieving a weighted F1-score of 0.92 versus 0.89, and was adopted for subsequent analyses. Predicted severity labels were consolidated into three categories to ensure sufficient sample sizes: Severe, Minor, and Enhancement (see Table 2 for details). The model not only exceeded the standard reliability benchmark of 0.85 but also surpassed the 0.90 threshold often applied in mission-critical applications (Yacouby and Axman 2020), confirming its suitability for downstream prediction tasks. In addition to these metrics, example classifications were manually spot-checked to ensure they were appropriate for public health software and have displayed exemplars in Table 2.

**Table 2:** Severity levels and definitions for bug classification.

Severity level	Definition	Example	Consolidated severity level
Blocker	A defect that completely prevents the system or a core feature from functioning. These bugs must be resolved before release or further development.	[Security] ensure getobject is at least v 1.0.0 due to security vulnerability.	Severe
Critical	A severe bug that leads to system crashes, data corruption, or security breaches. It poses high risk and requires immediate attention.	Remove accidentally committed print statement.	
Major	A defect that significantly impairs essential functionality but does not entirely block system operation. Often affects primary user tasks or workflows.	TRUNK-6274: Fix Issues with Hibernate Envers Audit Table Generation for Specific Tables.	
Minor	A bug that causes limited loss of functionality in non-critical features. These issues do not significantly impact user productivity or system stability.	fixing typo on readme.	Minor
Normal	A standard bug with minimal impact, often involving minor usability problems or less visible UI inconsistencies. Does not affect core functionality.	Clean up some UserES usages.	
Enhancement	Not a bug. Represents suggestions for new features, optimizations, or design improvements beyond the current implementation. No functionality is broken.	Add a consent form on Resilience Message flow.	Enhancement

Note: Severity level definitions were derived by the authors based on two bug-classification datasets (E1.csv and E2.csv) provided in the *bugClassificationSystem* repository. These datasets include bug descriptions and their corresponding classification labels, from which the operational definitions were summarized.

### Analytical approach

To assess differences in bug resolution dynamics, a multi-level modeling strategy tailored to the distributional properties of the outcome variables was adopted.

For each model, the mean, variance, and proportion of zero counts were computed to evaluate two key assumptions underlying standard Poisson regression: equality of the mean and variance, and absence of excess zeros. These diagnostics revealed substantial overdispersion across nearly all strata (variance-to-mean ratios well above 1) and pronounced zero inflation for severe bugs, particularly among revenue-driven organizations and projects maintained by donor-driven organizations. This indicates that a Poisson model would underestimate uncertainty and inflate Type I error rates. Based on these distributional checks, count regression models were selected according to the dominant data-generating process in each setting: Negative binomial models (NegBin) were used when overdispersion was present without excessive zeros; zero-inflated Poisson (ZIP) or zero-inflated negative binomial (ZINB) models were used when both overdispersion and a high proportion of zero weeks were observed.

Then, ITS regression models were estimated using weekly counts of closed bugs as the outcome, with the active bug load (measured in bug-days) included as an offset to model bug closure rates, while accounting for repository-level heterogeneity. Each model includes terms for baseline trend, level change at the intervention point, and post-intervention trend change. To accommodate heterogeneous intervention effects and respect the distinct distributional patterns identified in the diagnostic phase, the models were further stratified by company type and severity level. Results are reported as Incidence Rate Ratios (IRR).

Finally, to complement aggregate count-based analyses with individual-level resolution dynamics, Cox proportional hazards models were conducted to estimate the hazard of bug closure as a function of company type, severity, and intervention period. The survival models account for right-censoring of unresolved bugs and provide an alternative perspective on resolution efficiency that is independent of weekly aggregation. Together, the ITS count models and survival analyses capture both changes in overall bug resolution volume and shifts in the speed of resolving individual bugs around the funding freeze. All statistical analyses were conducted using R (version 4.4.1) in RStudio (Version 2024.04.2 + 764).

## Analysis and results

### Descriptive analysis

Across the eight selected digital public goods repositories, a total of 7922 issues and pull requests during the 2-year study window were identified. Among these, the majority were categorized as Minor (83%), far exceeding Enhancements (16%) and the relatively small share of Severe issues (1%–2%). Donor-driven and hybrid organizations achieved similarly high closure rates (86%), while revenue-driven entities accounted for 69%, lagging behind the other two types. Repository histories also differed; for example, some projects had bug records dating back to 2012 (e.g. OpenMRS, Bahmni), while others (e.g. Google’s Open Health Stack) were established as recently as 2020 (see Table 3).

**Table 3:** Descriptive statistics between June 2023 and June 2025.

	All/Pooled	Revenue-driven	Donor-driven	Hybrid
Number of Repositories	8	1	4	3
Bug Count, N (%)				
Total Bugs	7922	895	2220	4807
Severe	99 (1.25)	14 (1.56)	21 (0.95)	64 (1.33)
Minor	6558 (82.78)	730 (81.56)	1857 (83.65)	3971 (82.61)
Enhancement	1265 (15.97)	151 (16.87)	342 (15.41)	772 (16.06)
Bug Closed, N (%)	6655 (84.00)	621 (69.39)	1916 (86.31)	4118 (85.67)
First bug reported	–	March, 2020	August, 2012	March, 2012

The temporal analysis revealed substantial variation in bug load and management practices. Fig. 1 shows that, within each organizational type, the weekly trajectories of new issues, resolved issues, and closure rates tended to follow broadly similar patterns, indicating that closure rate fluctuations primarily reflected the balance between reporting and resolution rather than divergent underlying activity trends. However, distinct patterns emerged across organization types. Revenue-driven organizations exhibited the most volatile closure rates, with frequent short-term spikes and pronounced declines after mid-2024. In contrast, donor-driven organizations showed comparatively stable performance, maintaining closure rates within the 2%–10% range with modest variability. Hybrid organizations occupied a middle ground, displaying high overall closure rates (typically 5%–20%) and moderate week-to-week fluctuations.

**Figure 1:**
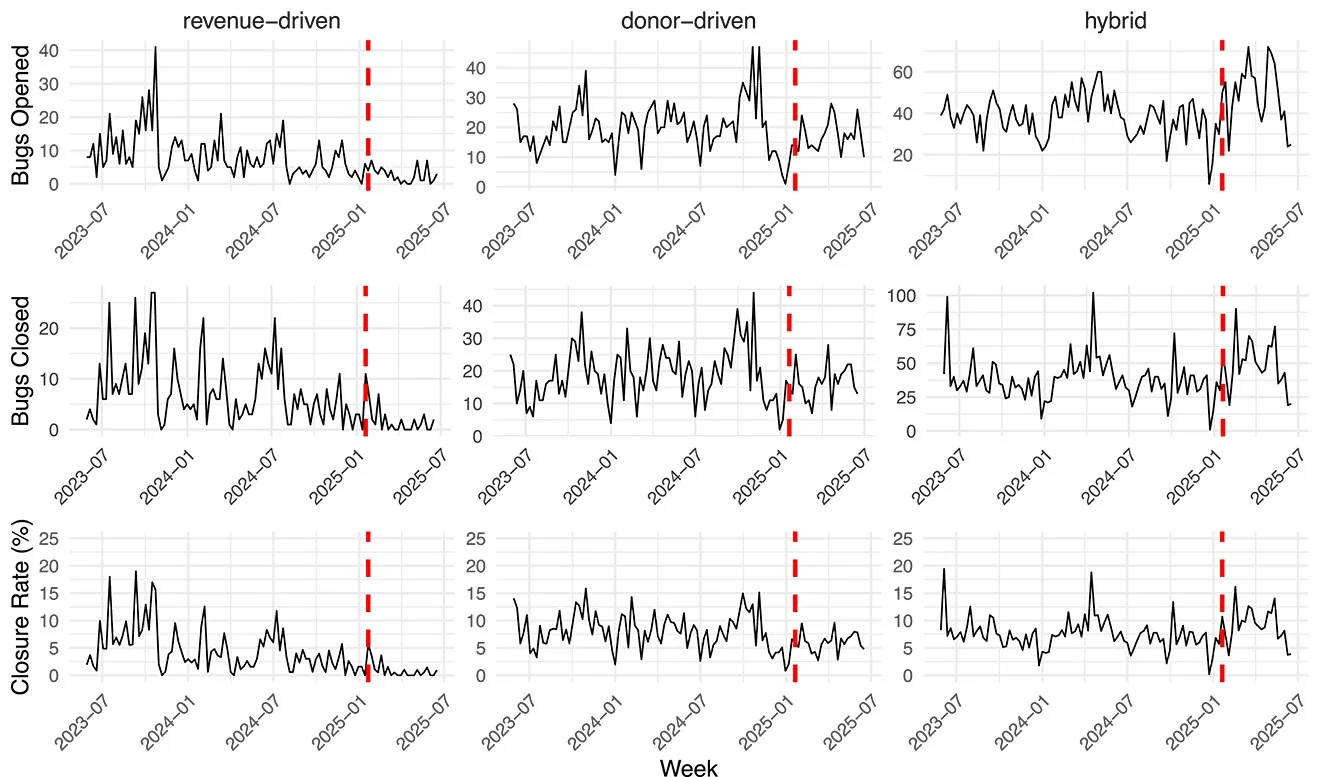
Weekly trajectories of bugs opened, closed, and closure rates by organization type (June 2023–June 2025).

The intervention marked by the January 2025 vertical line appears to have had immediate impacts across all organization types: revenue-driven organizations experienced a clear downward trend in closure rates; donor-driven and hybrid organizations responded more moderately; hybrid organizations exhibited a distinct post-intervention pattern, with closure rates rising initially and then declining within the six months after the intervention.

### Interrupted time series analysis

To assess temporal trends in bug resolution and changes associated with the intervention, ITS regression models were estimated with weekly counts of bugs closed as the dependent variable. An unadjusted regression model was first estimated to examine weekly bug closure rates over time. The model included a continuous time variable to capture the underlying weekly trend, a binary indicator marking the period after January 20, 2025, and an additional term representing the change in trend following the intervention. An offset for the log of weekly bug-days was incorporated to account for differences in exposure across weeks.

Since hybrid organizations exhibited a significant slowdown in bug resolution following the funding freeze when monthly fixed effects were included, an offset for the log of weekly bug-days was included to account for variation in exposure across weeks. Models including monthly fixed effects are presented as the main results, as they provide a more conservative specification and control for potential seasonal variation in issue reporting and developer activity. This approach reduces the likelihood that observed changes around January 2025 reflect routine seasonal fluctuations, such as holiday slowdowns and ramp ups.

Table 4 presents the unadjusted model results. In these models, *time* captures the underlying pre-intervention trend, *intervention* represents the immediate level change at the time of the funding freeze, and *time after intervention* reflects the change in trend following the intervention. In the pooled analysis, the intervention was associated with a statistically significant 25% increase in bugs closed per week (IRR = 1.25, *P* < .05), which was primarily driven by hybrid organizations.

**Table 4:** Unadjusted model – Bugs closed: Time series.

	All/Pooled	Revenue-driven	Donor-driven	Hybrid
Time	0.99 (0.00)^***^	0.98 (0.00)^***^	1.00 (0.00)^**^	1.00 (0.00)^***^
Intervention	1.25 (0.15)^**^	1.32 (0.39)	0.76 (0.19)	1.67 (0.16)^***^
Time after intervention	0.99 (0.01)	0.89 (0.03)^***^	1.01 (0.01)	0.99 (0.01)
Number of observations	972	324	324	324
Regression model	NegBin	ZINB	NegBin	NegBin

Note: *P* < .10^*^; *P* < .05^**^; *P* < .01^***^.

More specifically, revenue-driven organizations experienced a statistically significant post-intervention slowdown of 11% per week (IRR = 0.89, *P* < .01). Donor-driven organizations showed no statistically significant immediate level change or post-intervention trend change. Among hybrid organizations, the intervention was associated with a statistically significant and substantial immediate increase in bug closures of 67% (IRR = 1.67, *P* < .01).

Table 5 presents the adjusted regression models of weekly bugs closed, which analyze the variables across severity categories within each company type. Compared to the unadjusted model results (Table 4), incorporating severity and monthly fixed effects provides a more detailed interpretation of closure patterns.

**Table 5:** Adjusted model – Bugs closed: Time series.

	All/Pooled	Revenue-driven	Donor-driven	Hybrid
Time	0.99 (0.00)^***^	0.99 (0.00)^***^	1.00 (0.00)^***^	1.00 (0.00)^**^
Intervention	1.20 (0.16)	1.35 (0.42)	0.74 (0.23)	1.71 (0.23)^**^
Time after intervention	0.98 (0.01)^**^	0.90 (0.04)^***^	1.01 (0.02)	0.95 (0.02)^***^
Severity				
Minor	1.52 (0.13)^***^	1.06 (0.32)	2.32 (0.26)^***^	1.33 (0.17)^*^
Enhancement	1.41 (0.14)^**^	0.95 (0.33)	1.92 (0.27)^**^	1.37 (0.18)^*^
Number of observations	972	324	324	324
Regression model	NegBin	ZINB	NegBin	NegBin

Note: *P* < .10^*^; *P* < .05 ^**^; *P* < .01^***^.

In the pooled model, the overall temporal trend remained negative and significant (IRR = 0.99, *P* < .01), indicating that bugs were being resolved more slowly over time, consistent with the unadjusted results. A significant post-intervention slowdown was also observed (IRR = 0.98, *P* < .05), indicating a gradual decline in closure rates during the post-intervention period. Severity also played an important role: compared to severe issues, Minor (IRR = 1.52, *P* < .01) and Enhancement (IRR = 1.41, *P* < .05) bugs were resolved significantly faster.

Because the number of severe bugs is very small, the corresponding estimates have wide confidence intervals and should be interpreted with caution. For lower-severity bugs (minor and enhancement), donor-driven and hybrid organizations resolved these issues substantially faster than severe bugs (IRR = 1.37–2.32, all *P* < .10), whereas revenue-driven organizations showed non-significant differences by severity. Fig. 2 visualizes these adjusted results by displaying the estimated coefficients for the immediate and post-intervention effects across organization types and severity levels.

**Figure 2:**
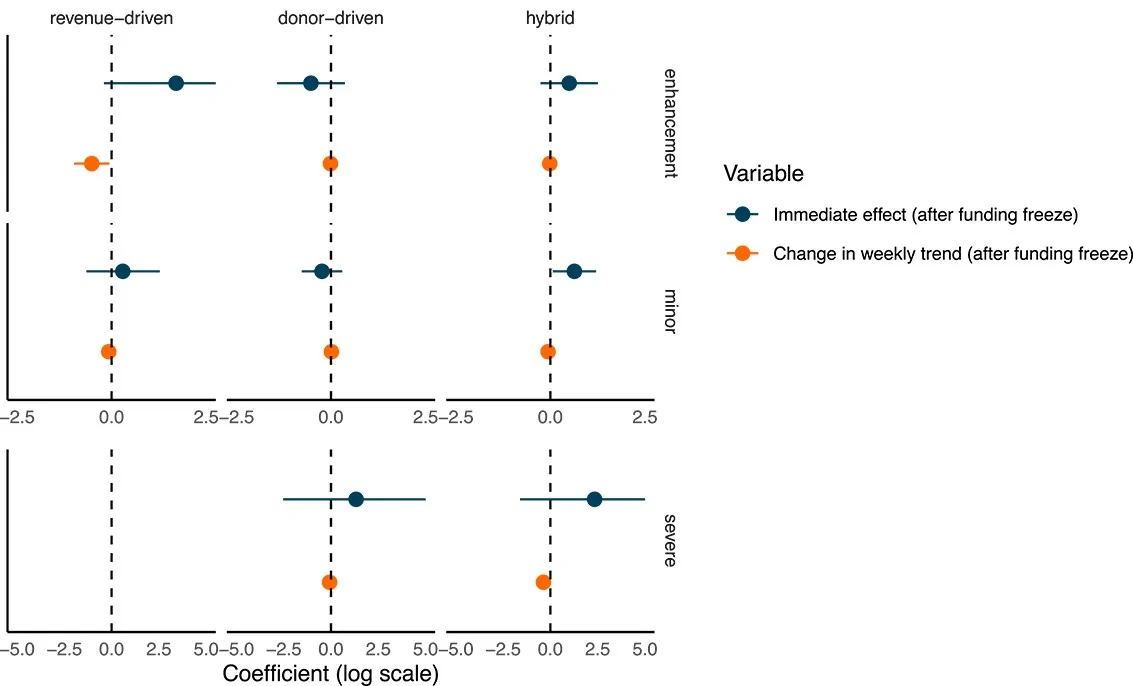
Estimated intervention and post-intervention effects on bug closures by organization type and severity.

The organizational sub-models reveal divergent strategies once severity is considered. Revenue-driven organizations displayed mixed responses by severity (Table 6). Severe bugs showed no significant time or intervention effects (IRR = 1.97 × 10^21^ for immediate intervention effect; this estimate is not interpreted due to sparse data). Minor issues showed no statistically significant immediate intervention effect, but exhibited a significant 7% weekly post-intervention slowdown (IRR = 0.93, *P* < .10). Enhancement issues responded more strongly to the intervention (IRR = 5.50, *P* < .10), but experienced a significant 41% weekly post-intervention decline (IRR = 0.59, *P* < .05), indicating challenges in sustaining resolution rates.

**Table 6:** Adjusted model – Bugs closed: Time series (by severity level) – Revenue-driven.

	Severe	Minor	Enhancement
Time	1.01 (0.02)	0.98 (0.00)^***^	0.99 (0.00)^*^
Intervention	1.97 × 10^21^ (1.24 × 10^4^)	1.34 (0.51)	5.50 (0.97)^*^
Time after intervention	0.00 (4.12 × 10^3^)	0.93 (0.04)^*^	0.59 (0.24)^**^
Number of observations	108	108	108
Regression model	ZIP	NegBin	NegBin

Note: *P* < .10^*^; *P* < .05 ^**^; *P* < .01^***^.

By contrast, donor-driven organizations showed no statistically significant change across any severity level (Table 7). Although the estimated IRRs for the time and intervention terms ranged from 0.59 to 3.54, none differed significantly from the null and therefore are not interpreted.

**Table 7:** Adjusted model – Bugs closed: Time series (by severity level) – Donor-driven.

	Severe	Minor	Enhancement
Time	0.97 (0.01)^**^	0.99 (0.00)^***^	1.00 (0.00)
Intervention	3.54 (2.03)	0.79 (0.27)	0.59 (0.46)
Time after intervention	0.93 (0.15)	1.01 (0.02)	0.99 (0.02)
Number of observations	108	108	108
Regression model	ZIP	NegBin	NegBin

Note: *P* < .10^*^; *P* < .05 ^**^; *P* < .01^***^.

For hybrid organizations, intervention effects differed by severity level (Table 8). Only Minor bugs exhibited a statistically significant immediate intervention effect, with an 89% increase in closures (IRR = 1.89, *P* < .05). Post-intervention trends showed significant declines for Severe (IRR = 0.66, *P* < .01) and Minor bugs (IRR = 0.94, *P* < .01).

**Table 8:** Adjusted model – Bugs closed: Time series (by severity level) – Hybrid.

	Severe	Minor	Enhancement
Time	1.00 (0.01)	1.00 (0.00)	0.99 (0.00)^***^
Intervention	5.70 (1.61)	1.89 (0.29)^**^	1.65 (0.39)
Time after intervention	0.66 (0.16)^***^	0.94 (0.02)^***^	0.98 (0.03)
Number of observations	108	108	108
Regression model	ZINB	NegBin	NegBin

Note: *P* < .10^*^; *P* < .05^**^; *P* < .01^***^.

Taken together, the adjusted results extend the findings of the unadjusted model, demonstrating that immediate intervention effects not only differed across organizational types but also varied in their long-term sustainability. Revenue-driven organizations exhibited significant intervention-related changes for Minor and Enhancement bugs, donor-driven organizations showed no statistically significant intervention effects or post-intervention trends, and hybrid organizations demonstrated a significant immediate increase only for Minor bug closures together with significant post-intervention declines for Severe and Minor bugs. The explicit inclusion of severity emphasizes that organizational strategies differ by bug type; severe issues are generally resistant to rapid resolution and exhibit unstable estimates due to sparse data.

### Survival analysis

Cox proportional hazards models were also applied to estimate the likelihood of bug resolution across organizational types while accounting for censoring.

The proportional hazards assumption was evaluated using Schoenfeld residual tests. For donor-driven and revenue-driven organizations, main indicators show no violation (*P* > .1), supporting the use of a standard Cox model. For hybrid organizations, the proportional hazards assumption was not satisfied when pre- and post-intervention periods were modeled jointly. The effects of key covariates varied across the two periods, violating the core assumption of time-invariant hazards. To address this issue, separate Cox models were estimated for the pre-intervention and post-intervention periods for hybrid organizations.

Additional diagnostic procedures were conducted to assess model adequacy. Martingale residuals showed patterns consistent with the Schoenfeld tests, and Cox–Snell residual plots closely followed the 45° reference line, indicating acceptable overall fit. Influence diagnostics based on dfbeta and deviance residuals identified a greater number of influential observations among hybrid organizations (maximum |dfbeta| = 0.06; 582 observations exceeding the dfbeta threshold) than among donor-driven (maximum |dfbeta| = 0.14; 232 observations) or revenue-driven organizations (maximum |dfbeta| = 0.06; 172 observations). However, the maximum dfbeta values remained small across all groups, suggesting that no individual observation exerted undue influence on the estimated coefficients.

For revenue-driven organizations, the intervention produced the strongest effect. The post-intervention period was associated with a substantial reduction in the hazard of bug resolution (HR = 0.54, *P* = .02), indicating that bugs became significantly less likely to be resolved after the intervention. This pattern aligns with the time series analysis, which showed a pronounced short-term spike in closures followed by a rapid slowdown. Repository age exhibited a marginally negative association with resolution (HR = 0.49, *P* = .09). Severity level and total bug volume were not significant predictors.

For donor-driven organizations, the intervention effect was not statistically significant, no evidence was found that the intervention changed the hazard of bug resolution. Repository age was positively associated with bug resolution (HR = 1.87, *P* < .05), whereas severity level and total bug volume were not statistically significant predictors.

For hybrid organizations, distinct patterns emerged across the two periods. Before the intervention, total bug volume was positively associated with the likelihood of resolution (HR = 1.26, *P* < .01), indicating that repositories with larger bug volumes tended to resolve bugs more quickly. Severity was not a statistically significant predictor, and repository age showed a significant positive association with bug resolution (HR = 1.45, *P* < .01). After the intervention, the effect of total bug volume became stronger (HR = 1.70, *P* < .01). Repository age also remained positively associated with bug resolution (HR = 3.13, *P* < .01), whereas severity continued to show no statistically significant association with the hazard of bug closure.

Figs 3 and 4 visualize the Cox proportional hazards results. Fig. 3 presents results stratified by company type. Fig. 4 further stratifies these patterns by both company type and severity.

**Figure 3:**
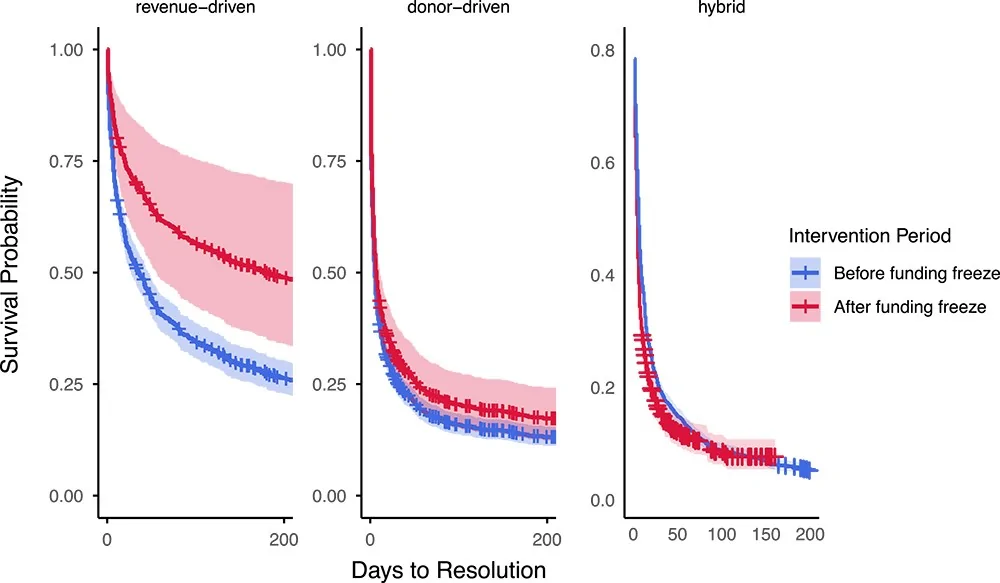
Cox proportional hazards survival curves for bug resolution by organization type.

**Figure 4:**
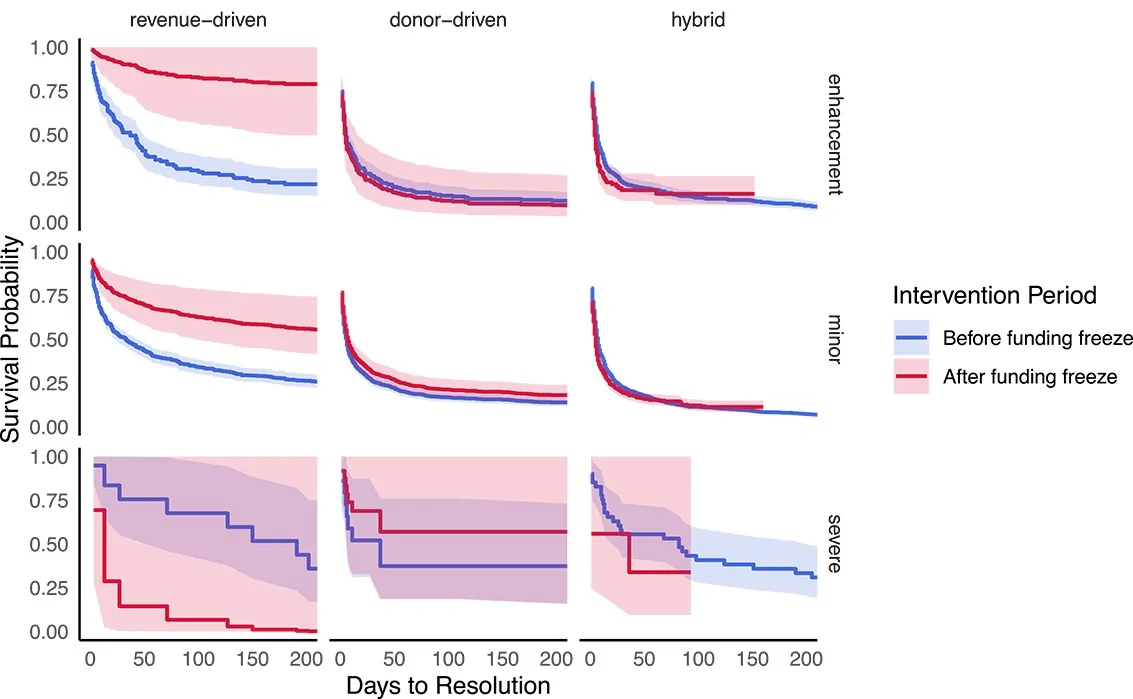
Cox proportional hazard curves by organization type and bug severity.

## Discussion

This study systematically examined how an external shock, the January 2025 USAID funding freeze, affected bug-maintenance efficiency in free, open-source digital public goods for community health workers and electronic health records in LMICs. Using descriptive statistics, ITS regression analysis and Cox survival modeling, the study found that although all organizational types exhibited varying degrees of responsiveness, their capacity to sustain or recover bug resolution performance diverged markedly. Revenue-driven entities exhibited no immediate drop following the intervention; however, they experienced a statistically significant decline of ~10% per week over the post-intervention period, demonstrating fragility under sustained fiscal uncertainty. Donor-driven organizations showed no statistically significant change in bug resolution time, suggesting relative stability following the funding freeze. Hybrid organizations showed the ability to buffer the immediate shock, followed by a significant weekly decline in overall closure rates.

These organizational effects are further disaggregated by bug severity. Enhancement bugs are often associated with feature requests, whereas minor bugs reflect routine maintenance; by contrast, severe bugs threaten core system functionality and are generally expected to receive priority regardless of funding conditions. Revenue-driven organizations exhibited post-intervention slowdowns primarily for minor and enhancement issues. This result should be read in light of the fact that the revenue-driven organization developed Open Health Stack in partnership with USAID and WHO and the uncertainty of that partnership was affected by the funding freeze (Google 2023; USAID Center for Innovation and Impact 2023). Among donor-driven organizations, no statistically significant intervention effects were observed, suggesting they remained resilient in their processes despite changes in the funding environment. Among hybrid organizations, minor bugs showed a statistically significant immediate increase in closures following the intervention, while enhancement bugs did not exhibit comparable immediate effects. Taken together, these findings suggest that funding disruptions could influence the composition as well as the overall volume of maintenance activity, although conclusions regarding the treatment of severe bugs should be interpreted cautiously given their low frequency and imprecise estimates.

Project life-cycle differences may help shape these dynamics. Repositories in this study varied substantially in age, with some dating back to 2012 and some established as recently as 2020; older projects are more likely to carry technical debt or long-standing bugs, which can influence the timing and likelihood of bug resolution and should be considered.

The results resonate with literature on FOSS sustainability and funding. Research has long highlighted that resource constraints pose significant challenges for open-source maintenance, particularly in public-interest software (Sonabend et al. 2024). The organizational diversity we observe, especially the strong performance of hybrid organizations, aligns with arguments that hybrid organizational models, which blend social mission with market logics, tend to be more resilient and sustainable, as demonstrated in studies of hybrid FOSS governance structures (Dorobat et al. 2024; Carballo 2023) and in broader analyses of social-enterprise hybridity (Fu and Yan 2025; Mikołajczak 2020). Moreover, others have stressed that public funding’s impact depends on project governance, life-cycle stage, and social structure (Fu and Yan 2025), which is consistent with our finding that the underlying funding mechanism strongly mediated the effect of the external shock.

The findings also challenge some prevailing views. While some scholars suggest that purely commercial open-source models are more sustainable because of their ability to monetize services (Li et al. 2024), our data showed that under conditions of funding volatility, revenue-driven entities can be the most vulnerable and least stable. In our case, the revenue-driven software was explicitly designed for use in LMICs. When this external funding became uncertain, the private-sector actor ceased active engagement: the external partners driving the collaboration (e.g. USAID and WHO staff) were no longer available, removing the operational and mission-driven rationale for continued involvement (Google 2023; USAID Center for Innovation and Impact 2023). As a result, despite their financial agility in stable environments, these market-driven OSS firms experienced rapid erosion of activity once external incentives disappeared. This finding raises concerns about relying exclusively on such actors for critical global public-health infrastructure, where resilience to funding fluctuations is essential.

This study has some limitations. First, the study lacked fine-grained control for important covariates such as contributor network structure, team size, and technical debt, which may mediate an organization’s adaptive capacity. Second, although the DistilBERT classifier achieved a high weighted F1 score on the training data, potential domain shift between the general GitHub issue datasets used for training and the specific context of global health software cannot be fully ruled out. Third, the exact level of USAID funding received by each project could not be verified at the repository level. However, placebo tests were conducted by assigning hypothetical intervention dates prior to the January 2025 funding freeze (April, July, and October 2024). Results show that only the specification aligned with October 2024, which coincides with the start of the U.S. fiscal year and the period immediately following the funding disruption, produced a substantial share of statistically significant changes in bug resolution activity (>50%). In contrast, earlier placebo dates yielded far fewer significant effects (˂25%). This pattern suggests that the observed decline in bug closure is unlikely to be driven by seasonal or other unrelated trends and is instead consistent with the USAID funding disruption. Fourth, the analysis focuses on issue- and pull-request–level data but does not examine developer-level behaviors, such as commit history or collaboration patterns, which could provide richer insight into resilience mechanisms. Fifth, this study used an ITS design without untreated comparison groups. Although a controlled design would strengthen causal inference, all digital public goods included in this study were embedded within the same USAID-supported global health ecosystem, which made the identification of a valid contemporaneous control group infeasible. Sixth, although repository-level random effects were incorporated to account for heterogeneity, the number of repositories within each organizational category was limited, particularly for the revenue-driven (n = 1) and hybrid groups (n = 3). Therefore, the findings should be interpreted as evidence from a small but policy-relevant sample.

Finally, this study does not explicitly account for the rapid adoption of generative artificial intelligence (AI) tools in software development workflows beginning in late 2023. The increasing accessibility of AI-assisted coding tools may have improved developer productivity and accelerated bug identification and resolution across repositories during the study period. To the extent that these tools reduced maintenance burdens, they may have partially offset the negative effects of the USAID funding freeze. As a result, the estimated declines in bug resolution efficiency may be conservative. If AI adoption did enhance baseline productivity, the true impact of the funding disruption on maintenance dynamics may be larger than what is captured in this analysis.

In conclusion, external funding shocks act as stress tests for the resilience of digital public goods ecosystems. The study highlights that not all organizations are equally equipped to absorb and adapt to fiscal uncertainty: hybrid structures appear capable of sustaining overall maintenance activity under fiscal uncertainty, showing resilience at the time of the shock, but deteriorating over time; donor-driven organizations suggest institutional stability, experiencing only transient disruption before returning to baseline performance; while revenue-driven organizations may reduce their social digital public good purpose when the partnership is uncertain. Building more resilient digital public goods requires not only technical robustness, but also organizational designs, especially governance models, that combine mission-driven purpose with adaptive flexibility.

## Data Availability

The data are available to download from Github from each repository.
